# Predictors for improvement in personality functioning during outpatient psychotherapy: A machine learning approach within a psychodynamic psychotherapy sample

**DOI:** 10.1192/j.eurpsy.2024.1780

**Published:** 2024-11-15

**Authors:** I. Dönnhoff, D. Kindermann, S. Stahl-Toyota, J. Nowak, M. Orth, H.-C. Friederich, C. Nikendei

**Affiliations:** Centre for Psychosocial Medicine, Department of General Internal Medicine and Psychosomatics, University Hospital Heidelberg, Heidelberg, Germany

**Keywords:** personality functioning, machine learning, missing data analysis in machine learning, psychotherapy success

## Abstract

**Background:**

Since its introduction in the diagnostic manuals DSM-5 and ICD-11, the construct of personality functioning has gained increasing attention. However, it remains unclear which factors might predict improvement in personality functioning.

**Methods:**

We examined a sample of 648 completed psychodynamic psychotherapies conducted by 172 therapists at the Heidelberg Institute for Psychotherapy. A machine learning approach was used to filter for variables that are relevant for the prediction of the improvement of personality functioning from a broad data set of variables collected at the beginning of each psychodynamic psychotherapy.

**Results:**

On average, we found an improvement of 0.24 (SD = 0.48) in the OPD-SQ. This corresponds to a medium effect in the improvement of personality functioning. Patients with initially high impairment experienced particularly large improvements. Overall, we found a large number of variables that proved to be predictive for the improvement of personality functioning. Limitations in social activity due to physical and emotional problems proved to be one of the most important predictors of improvement. Most of the effect sizes were small.

**Conclusions:**

Overall, the improvement in personality functioning during psychotherapy is determined more by the sum of a large number of small effects than by individual variables. In particular, variables that capture social areas of life proved to be robust predictors.

## Introduction

In their latest editions, both the diagnostical and statistical manual of diseases (DSM) and the international classification of diseases (ICD) have introduced a dimensional concept of personality disorders, measured in part by personality functioning [1, 2]. Historically, the concept of personality functioning was first introduced in the operationalized psychodynamic diagnosis (OPD) almost 30 years ago [3]. Based on psychoanalytic theory, especially ego psychology, it was used to describe psychological abilities of a person to cope with inner conflicts and interpersonal problems [3]. According to the, personality functioning can be described by four dimensions each subdivided with regard to the self and others: perception, regulation, communication and attachment capacity (Supplement A) [3]. A patient can be categorized into one of 4 levels depending on the severity and rigidity of their impairment in one subdimension [3, 4]: High, moderate, low level of integration or disintegration. Finally, an overall impression was made on the basis of these assessments and the focus of treatment was set on the basis of this assessment.

In 2013, the DSM took up a long-standing criticism of the existing model of personality disorders, as it was shown that only 50% of all personality disorders are represented by the existing categories [5, 6]. Furthermore research accumulated that personality disorders seemed to be based on intrapsychic and interpersonal impairment [7]. In developing a model for personality functioning, the DSM-5 task force was inspired by existing models from psychoanalytic research [7]. One influential model was Kernberg’s model of personality organization, which encompassed identity, reality testing, defense, and object relations [5, 7, 8]. In this synthesis of the existing research situation, the DSM-5 task force decided to divide personality functioning into two basic dimensions, each with two subdimensions: the self with identity and self-direction and interpersonal relationships with empathy and closeness [1].

In 2021, the 11th revision of the ICD also introduced a dimensional model of personality disorders [2]. As in the AMPD of the DSM-5, the ICD-11 distinguishes between the self and interpersonal skills, but does not further divide them into two subdimensions. The ICD-11 also retains a distinction of impairment in 4 levels: severe, moderate and mild personality disorder and personality difficulty [5, 6, 9]. The ICD-11 added a new assessment of the chronicity and rigidity of impairments [9].

To summarize in both the DSM-5 and the ICD-11 personality functioning is considered to be an underlying construct of personality disorders. In the OPD, personality functioning is seen as a person’s psychological abilities that they develop over the course of their life to cope with inner conflicts and interpersonal problems. Thus, the conceptualization in the OPD includes most of the concepts from both the DSM-5 and ICD-11 (Supplement A) [3, 4, 10]. In fact, Zimmermann et al. could show that the global measurements of the OPD, the DSM-5 and the ICD-11 measure the same latent construct [10]. Recent studies have shown that personality functioning also varies in depressive disorders and anxiety disorders and can be used to predict how quickly a patient will improve in psychotherapy[11-13]. This suggests that personality functioning should not only be seen as a construct to capture personality disorders, but as a more general construct. Therefore, for this study, we use the OPD definition of personality functioning.

Along with this, research in recent years has shifted to view personality functioning as a dimension to be improved through psychotherapy: Several authors found that personality functioning can be improved by inpatient and outpatient psychotherapy [14-20]. However, only few studies have investigated the predictors of improvement in personality functioning: Flemming et al. found high attachment avoidance to be predictive of less improvement [21]. Kvarstein et al. found that borderline personality disorder was associated with greater improvements and older age with lower improvements in personality functioning [22].

Explorative, data-driven procedures, commonly described as machine learning, represent one possible approach to determine influencing factors of improvement in personality functioning [23-25]. In supervised machine learning, the mathematical algorithm attempts to develop a model that predicts the dependent variable with as little error as possible [25]. Depending on the structure of the data and the researcher’s objective, various mathematical algorithms can be used [25, 26]. However, this explorative, data-driven approach is rarely used in psychotherapy research, especially psychodynamic psychotherapy research [27, 28].

Considering the aspects mentioned above, the present study was designed to utilise machine learning to identify relevant variables that predict the improvement of personality functioning. For this purpose, we considered all variables that were routinely recorded at the beginning of psychotherapy at the Heidelberg Institute for Psychotherapy (HIP) as potential predictors [29]. Then, we used supervised learning algorithms to filter for variables that are relevant for predicting the improvement of personality functioning. Finally, we verified our results on a separate data set to assess generalizability.

## Methods

### Study design

This study involves an exploratory and retrospective analysis of routinely assessed longitudinal psychotherapy treatment data from the Heidelberg Institute for Psychotherapy (HIP), University of Heidelberg in Germany. The HIP is a training institute for psychodynamic therapists [29].

### Participants

#### Patients

This study is based on a sample of N_Patients_ = 648 completed outpatient psychodynamic psychotherapies. Age ranged from 18 to 76 years (M = 35.6, SD = 13.1). On average, patients received 53.7 sessions of psychotherapy (SD = 28.4, range: 1 to 120). The number of SKID-Diagnoses ranged from 0 to 10 (M = 2.4, SD = 1.6). Overall improvement in personality functioning ranged from -1.4 to 1.7. These psychotherapies took place between January 2013 and July 2021. Patients were included after providing written informed consent. To be included in the study, patients had to (1) be 18 years old, (2) speak German or English and (3) have had at least one diagnostic session with a therapist.

#### Therapists

All therapists were in training to become psychodynamic orientated therapists. N_Therapists_ = 172 therapists participated in this study. Therapists had to (1) either have a degree in psychology (M.Sc. or Ph.D.) or be a medical resident (MD) and (2) have at least 1.5 years of clinical experience. On average, each therapist treated approximately M = 9.4 patients (SD = 5.4). The treatment was supervised every fourth session by an experienced psychodynamic orientated therapist with at least five years of experience.

### Ethics

The study protocol was developed according to the Helsinki II declaration [30]. Prior to recruitment of patients and therapists, the study was approved by the independent ethics committee of the Medical Faculty of the Heidelberg University (S-195/2014). Written informed consent was obtained from all study participants.

### Procedure

#### Diagnostic assessment

Each patient took part in a clinical interview in an outpatient department to assess the indication for a psychodynamic psychotherapy [31]. In this interview, the patients were informed about the study and invited to participate. Written informed consent was then given. After the intake interview, patients answered sociodemographic and psychometric questionnaires and were invited to a standardised diagnostic interview [SCID-I and SCID-II; [32], German version: [33]] with a trained psychologist. Finally, the patient was referred to one of the study therapists.

#### Psychotherapy

The treatment took place once a week for 50 minutes and consisted of individual depth psychologically founded psychotherapy [34]. Treatment focuses on current psychosocial problem, which are worked through as re-actualized conflicts and results of structural deficiencies [34]. Mean number of session was 53.7 (SD = 28.4). German public health insurance covers the full cost of treatment if patients apply for a distinct number of sessions in advance (12, 24, 60 or 100 sessions). Before applying for a distinct number of psychotherapeutic sessions, patients attend up to seven diagnostic and preparatory sessions for diagnostic reasons. The number of sessions is agreed upon with the therapist. After the first therapy session, the therapists and patients were asked to complete psychometric questionnaires. At the end of the last psychotherapy session, both the patient and the therapist were asked to complete psychometric questionnaires once again.

### Instruments

Patients and therapists were asked to complete a total of 14 different psychometric questionnaires after the initial intake interview and the first requested session. Please see Supplement B for a description of all 14 questionnaires.

### Data analysis

**Figure 1.**
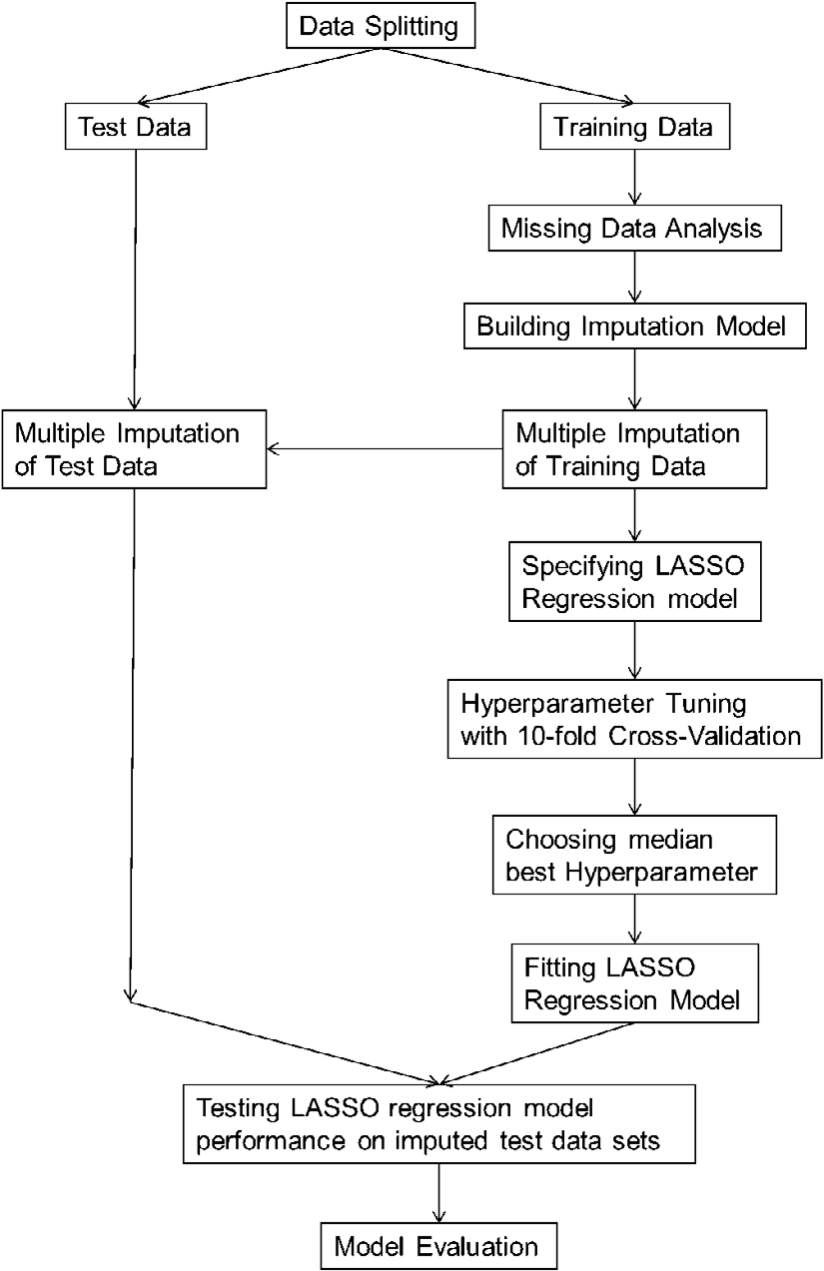
Data analysis‘ process.

The complete analysis was done using R version 4.3.1. [35]. The individual steps of the data analysis can be seen in Figure 1. We operationalized our target variable ‘improvement in personality functioning’ by subtracting the mean value of the OPD-SQ post-questionnaire from the mean value of the OPD-SQ pre-questionnaire. A positive difference value therefore indicates an improvement in personality functioning.

#### Data splitting

To allow an unbiased assessment of model performance, to check for overfitting and to only utilize training data for model development we first randomly divided the dataset into training and test data [25, 36]. Using the “Rsample” package [37], we split the data into 80% training (N_training_ = 518) and 20% test data (N_test_ = 130).

#### Missing data analysis (of training data)

We removed variables with more than 30 % missing values and an influx above 0.5 [36]. Our target variable ‘improvement of personality functioning’ had 42% missing values and in total the training data had 14.4% missing values. All missing values correlated significantly with each other. We concluded that it was a patient variable that generated the missing values. The missing values in our target variable ’improvement of personality functioning’ correlated with previous inpatient psychiatric treatment, with substance abuse disorder, with the way therapy ended (regular, terminated, prematurely ended), and negatively with school education. Since we recorded all these variables and were able to incorporate them into our imputation model, we assumed a ’missing at random’ process to be reasonable.

#### Building imputation model

We performed the multiple imputation using the ‘mice’ package [36, 38]. All variables with a correlation of at least 0.2 were used as predictors, resulting in a median of 28 predictors per variable. Sums and means of several scales were imputed using passive imputation, other variables were imputed with predictive mean matching [36]. Based on our considerations above, the end of therapy (completed, terminated, prematurely ended) was also added as a predictor for all variables. We first generated 10 imputed training data sets with 120 iterations of the algorithm. Plotting the means against the iteration number the streams intermingled freely with no definite trends [36]. Furthermore, density plots and scatter plots showed that the data could have come from real patients. We judged the imputation model as being good and used it to create 30 imputed training data sets each using 150 iterations of the algorithm. Using the same imputation model, we created 30 imputed test data sets.

#### Lasso regression model

*Predictors.* All sociodemographic variables and scales of the psychometric questionnaires that were available at the beginning of therapy were used as predictors.

*Hyperparameter Tuning and Selection.* We used the ‘glmnet’ package [39] and the ‘tidymodels’ [37] framework for hyperparameter tuning and all other following calculations. We opted for a lasso regression, as it uses soft thresholding to remove all variables with low regression coefficients from the model by setting the regression weight to zero [25]. The strength of the thresholding depends on a hyperparameter λ_penalty_ that we determined for all 30 imputed training data sets with 10-fold cross validation, using 2001 evenly distributed possible values for λ_penalty_ between 0 and 0.5 [25]. For each of the 30 imputed training data sets, we then selected the λ_penalty_ with the lowest cross validation mean absolute error (MAE_cv_).

*Model Performance.* Then, a final lasso regression model for each of the 30 training datasets was calculated using the median of the 30 selected λ_penalty_. To check for overfitting, we tested all 30 models on all 30 imputed test data sets. For each test data set, we predicted the improvement in personality functioning and calculated the MAE_test_. Then, we pooled the MAE_test_ using Rubin’s rule [36]. Overfitting was defined as MAE_test_ being two standard deviations above MAE_cv_.

*Model Evaluation.* For each variable, we calculated 2 parameters: the pooled regression weight according to Rubin’s rule [36] and the number of models in which the regression weight of the variable was set to zero. If the regression weight of a variable was set to zero in five or less models, the variable could be described as a robust predictor.

### Transparency

Our complete analysis and the analysis code can be found under the following DOI: doi.org/10.11588/data/50WFVL. To ensure the transparency of our evaluation, we adhere to the TRIPOD guidelines [40]. The datasets used and analysed during the present study cannot be shared due to restrictions by the Ethics Committee of the University of Heidelberg.

## Results

### Training and test sample description

The description of our training and test sample as well as the grand means of imputed training and test data sets can be seen in Table 1. On average, the test sample included a larger proportion of women and a higher number of previously utilised psychotherapeutic and psychiatric services. The training sample contained a higher proportion of depressive disorders, somatoform disorders, and eating disorders. In contrast, the test sample showed a higher proportion of personality disorders and substance abuse disorders. Both samples had a similar average level of personality functioning at the start of therapy. However, the improvement in personality functioning in the training sample was greater. Patients in the test sample terminated their treatments less frequently and brought them to a regular end more often. Furthermore, the overall mean value of the imputed data hardly differs from the values of the non-imputed data. Due to these differences between training and test data, it can be assumed that the model only receives little information about the test data from the training data set. Checking the performance on the test data set therefore represents a good test for overfitting.

**Table 1.** Sample description of training and test sample

	Training Data				Test Data			
Variable	M	SD	GM	MSD	M	SD	GM	MSD
Female	62 %	48 %	62 %	2 %	68 %	47 %	68 %	4 %
Age	35.6	13.10	35.60	0.63	35.73	13.18	35.94	1.25
Sessions applied for	60.53	28.4	60.3	1.52	57.13	28.29	56.40	2.7
Psychotropic medication	28 %	45 %	28 %	2 %	28 %	45 %	28 %	4 %
Past Psychotherapy	0.47	0.77	0.45	0.03	0.66	0.94	0.65	0.09
Inpatient Psychiatry	0.18	0.47	0.17	0.02	0.24	0.59	0.23	0.05
Pre OPD-SQ mean	1.60	0.52	1.60	0.02	1.60	0.52	1.61	0.05
Terminated Therapy	13 %	34 %	13 %	1 %	8 %	28 %	8 %	2 %
Completed Therapy	62 %	48 %	62 %	2 %	70 %	46 %	70 %	4 %
Premature Ended Therapy	11 %	32 %	11 %	1 %	8 %	28 %	08 %	2 %
Depression	76 %	43 %	76 %	2 %	72 %	45 %	72 %	4 %
Anxiety Disorder	48 %	50 %	48 %	2 %	48 %	50 %	48 %	4 %
Personality Disorder	19 %	40 %	19 %	2 %	25 %	44 %	25 %	4 %
Substance Abuse Disorder	12 %	32 %	12 %	1 %	18 %	39 %	18 %	3 %
Somatoform Disorder	12 %	32 %	12 %	1 %	8 %	27 %	8 %	2 %
Eating Disorder	14 %	35 %	14 %	2 %	8 %	28 %	8 %	2 %
PTSD	5 %	23 %	5 %	1 %	5 %	21 %	5 %	2 %
OPD-SQ Mean Difference	0.24	0.48	0.24	0.03	0.17	0.49	0.19	0.07

*Note:* M = Mean of Data without imputed data sets, SD = Standard Deviation of Data without imputated data sets, GM = Grand Mean of all imputed data sets, MSD = Standard Deviation of Means of imputed data sets. For Past Psychotherapy and Stationary Psychiatry values represent the mean number of previous treatments, respectively. Terminated, completed and prematurely ended therapies each indicate the proportion of patients who terminated therapy without the therapist’s consent, who completed therapy or who had to terminate therapy prematurely with the therapist’s consent.

### Evaluation of overfitting

**Figure 2.**
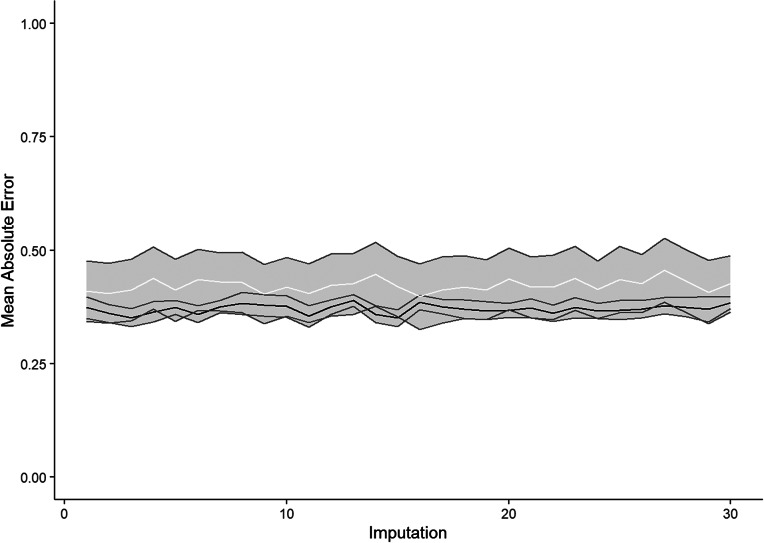
Comparison of cross-validation and test mean absolute error. *Note:* Black line represents Cross-validation errors and white line represents test errors. Grey area represents two standard errors, respectively.

Figure 2 shows the cross-validation and test MAE plotted against imputation. Across all imputations, the deviation of the test MAE is smaller than two standard errors of the test mean absolute error. Therefore, we assume that we have not overfitted our model. The mean cross-validation MAE is 0.37 (SE = 0.01) and the mean test MAE is 0.42 (SE = 0.04). This means that the prediction of the improvement in personality functioning by our model deviates from the actual improvement by a median of 0.37 in the training data set and by a median of 0.42 in the test data set.

### Evaluation of model error

**Figure 3.**
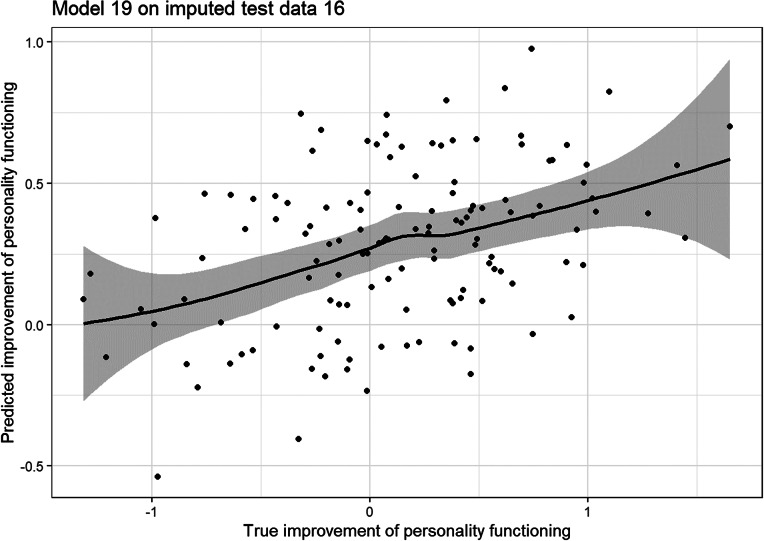
Example plot of model test errors.

Figure 3 shows an example of the performance of the final model on the test data. The model overestimates the improvement in personality functioning in patients who have not improved or even deteriorated. On the other hand, it underestimates the improvement of patients who improved significantly. Overall, however, there is a linear relationship between the prediction of the model and the actual improvement in the patient’s personality functioning (r_median_ = 0.391, r_mean_ = 0.386, r_min_ = 0.147, r_max_ = 0.568). Thus, the trend of the prediction corresponds to the actual improvement of the patient.

### Final model

#### Removed variables

The number of models a variable was removed from can be seen in Table 2. Eight variables were used in all models: The Intercept, pre OPD-SQ mean, the scale ‘limitation in social activities’ of the SF, the scales ‘Too caring’ of the IIP, the scale ‘Depression’ of the PHQ, the scale ‘Task’ of the WAI rated by the therapist, gender, and former outpatient psychiatric treatment. Furthermore, 6 variables were used in 29 of 30 models: the scales ‘Hard to be involved’ and ‘Hard to be supportive’ of the IIP, the subscale ‘Stress’ of the PHQ, former inpatient psychiatric treatment, the scale ‘Smoothness’ of the SE rated by the therapist, and retirement of the patient. In total we found 25 robust predictors of improvement in personality functioning, which can be seen in Table 2.

**Table 2.** Average penalized regression weights and number of times variable was removed

Variable	N_removed_	β_penalized_	SE_β_	λ	γ
Intercept*	0	0.24	0.02	1.00	1.00
Pre OPD-SQ mean*	0	0.16	0.06	0.93	0.95
SF – Lim. In social activities*	0	0.11	0.02	0.54	0.59
IIP – Too caring*	0	–0.07	0.03	0.56	0.60
DEQ – Self Criticism*	4	0.06	0.05	0.88	0.91
PHQ – Depression*	0	0.06	0.03	0.68	0.72
IIP – Hard to be involved*	1	–0.05	0.03	0.70	0.74
IIP – Hard to be supportive*	1	–0.05	0.03	0.61	0.65
PHQ – Stress*	1	0.05	0.03	0.72	0.76
WAI – Therapist – Task *	0	0.04	0.02	0.44	0.48
Past Inpatient Psychotherapy*	2	–0.04	0.03	0.59	0.63
OPD – Internal Attachment*	2	0.04	0.03	0.77	0.81
ECR-R – Anxiety*	3	–0.04	0.03	0.73	0.77
Gender*	0	0.04	0.02	0.42	0.46
Past Inpatient Psychiatry*	1	–0.03	0.03	0.66	0.70
APK – PCE*	3	0.03	0.03	0.57	0.61
SCL-K11	9	0.03	0.04	0.85	0.88
APK – ACE*	4	–0.03	0.03	0.71	0.75
Past Outpatient Psychiatry*	0	–0.03	0.02	0.48	0.53
BSS – 7 Days*	5	–0.03	0.03	0.58	0.62
PHQ – Anxiety*	5	–0.02	0.03	0.60	0.64
SEQ – Therapist – Smoothness*	1	0.02	0.02	0.48	0.52
Retirement*	1	–0.02	0.02	0.20	0.24
OPD – Self Perception	11	0.02	0.03	0.69	0.73
ECR-R – Avoidance*	3	0.02	0.02	0.48	0.52
Relationship*	3	–0.02	0.02	0.36	0.40
OPD – External Attachment	6	0.02	0.02	0.51	0.55
Obsessive-Compulsive Disorder	7	0.02	0.02	0.43	0.47
Past Outpatient Psychotherapy	7	0.02	0.02	0.48	0.53
Depression	9	0.01	0.02	0.32	0.36
SF – Role Lim. due phys. health	10	0.01	0.02	0.46	0.50
OQ – Social Role	8	–0.01	0.02	0.38	0.42
BSS – Last Year	10	0.01	0.02	0.42	0.46
SEQ – Patient – Depth*	5	–0.01	0.02	0.24	0.28
Children	10	–0.01	0.02	0.40	0.44
Disability Pension	10	–0.01	0.02	0.31	0.35
SF – Change in Health	9	0.01	0.02	0.39	0.43
Number of sick days	7	–0.01	0.02	0.34	0.38
OPD – Other Perception	17	0.01	0.02	0.52	0.56
Highest School Leaving Certificate	9	–0.01	0.02	0.34	0.38
PTSD	10	0.01	0.02	0.31	0.35
OPD – Other Regulation	14	0.01	0.02	0.49	0.53
IIP – Too dependent	10	–0.01	0.02	0.36	0.40
DEQ – Dependency	11	0.01	0.03	0.67	0.71
RequestedTherapyHours*	4	–0.01	0.02	0.22	0.26
BDI-II	19	0.01	0.02	0.50	0.54
Anxiety Disorder	10	–0.01	0.02	0.29	0.32
OPD – Internal Communication	16	0.01	0.02	0.40	0.44
Divorced	10	0.01	0.02	0.19	0.23
Number of Relatives to care for	14	0.01	0.02	0.30	0.34
SEQ – Patient – Smoothness	12	0.01	0.02	0.27	0.31
Psychotropic medication	13	0.01	0.02	0.20	0.24
WAI – Patient – Bond	18	0.01	0.02	0.32	0.36
IIP – Hard to be sociable	17	0.01	0.02	0.43	0.48
Eating disorder	11	–0.01	0.02	0.18	0.22
Number of doctor visits	12	0.01	0.02	0.20	0.24
SF – Bodily Pain	14	0.01	0.02	0.19	0.23
SF – General Mental Health	20	–0.01	0.02	0.36	0.41
OPD – Self Regulation	21	0.01	0.02	0.39	0.43
IIP – Hard to be supportive	19	–0.01	0.02	0.29	0.33
IIP – Too open	19	0.00	0.02	0.23	0.27
Personality Disorder	12	0.00	0.02	0.12	0.15
Married	12	0.00	0.02	0.15	0.19
SF – Vitality	20	0.00	0.02	0.25	0.29
OPD – External Communication	20	0.00	0.02	0.39	0.43
OQ – Interpersonal Relations	19	0.00	0.02	0.28	0.32
SF – Lim. In phys. activities	22	0.00	0.02	0.20	0.23
SF – General Health Perception	15	0.00	0.02	0.08	0.12
Concurrent outpatient Psychiatry	14	0.00	0.02	0.11	0.15
GAF	22	0.00	0.02	0.21	0.24
WAI – Therapist – Goal	21	0.00	0.02	0.12	0.16
IIP – Too aggressive	13	0.00	0.02	0.25	0.29
OQ – Subjective Discomfort	27	0.00	0.02	0.26	0.30
SEQ – Therapist – Depth	14	0.00	0.02	0.24	0.28
Adjustment disorder	14	0.00	0.02	0.11	0.15
Substance abuse disorder	18	0.00	0.02	0.13	0.17
SF – Role Lim. due emot. Prob.	22	0.00	0.02	0.17	0.20
Degree of employment	17	0.00	0.02	0.18	0.22
WAI – Patient – Task	20	0.00	0.02	0.12	0.16
Past Psychotropic medication	19	0.00	0.02	0.08	0.12
Somatoform Disorder	19	0.00	0.02	0.10	0.14
Somatoform Pain Disorder	16	0.00	0.02	0.09	0.13
WAI – Therapist – Bond	21	0.00	0.02	0.02	0.05
Highest Professional Degree	20	0.00	0.02	0.12	0.15
PHQ – Somatic Symptoms	17	0.00	0.02	0.25	0.29
WAI – Patient – Goal	13	0.00	0.02	0.24	0.28
Household Type	16	0.00	0.02	0.10	0.13
Age	26	0.00	0.02	0.08	0.12

*Note:* N_removed_ = Number of models this variable was removed from. β_penalized_ = Grand mean of the penalized regression coefficient. SE_β_ = Rubin’s rule pooled standard error of the penalized regression coefficient. λ = Proportion of Variation of β_penalized_ attributable to missing data. γ = Fraction of information missing about β_Penalized_ due to missing data. Caution: As β_penalized_ is not t-distributed significance calculation cannot be done. Gender: men = 1, women = 2. All variables were standardised. This means that the β_penalized_ indicates by how much the improvement in personality functioning changes if the respective variable is increased by 1 standard deviation. Variables which were set to zero in five or less models, and defined as robust, are marked by a*.

#### Influence of variables

The pooled penalized lasso regression coefficients of a variable on the improvement in personality functioning can also be seen in Table 2. The absolute size of the pooled penalized lasso regression coefficients correlates with the number of models in which a variable was removed (r = -0.588).

#### Influence of missing values on results

Table 2 also shows how much variance was generated by the missing values or how much information was lost. A lot of variance was generated by the missing values, especially for the variables with a large pooled penalized lasso regression coefficient.

## Discussion

We used machine learning to filter for variables that predicted the improvement of personality functioning. Then, we checked the model for overfitting on a separate test data set. There was no overfitting as the final model showed a comparable performance to the training data set. Personality functioning improved on average by 0.24 (SD = 0.48) points on the OPD-SQ scale from an initial average value of 1.60 [41]. This corresponds to a medium effect size [42]. Compared to the development study of the OPD-SQ, this improvement in our sample corresponds to two thirds of what would be required to reach the level of personality functioning of a healthy control sample [41]. In total, we found 25 robust predictors of improvement of personality functioning with mostly small effect sizes. This means that the improvement in personality functioning is primarily explained on a multi-causal basis. In the following, a sample of these 25 predictors are discussed.

The variable with the greatest predictive power is the initial impairment of personality functioning. Patients with greater impairment at the beginning of outpatient psychotherapy experience greater improvement in personality functioning during psychotherapy. This result is in line with the findings of Kvarstein et al., who also found that personality functioning can improve especially for severely impaired patients, such as borderline personality disorder patients [22]. Although we did not find a connection between treatment discontinuation and personality functioning in our study, this has been shown in other works [15, 43]. If it is possible to motivate these patients to complete psychotherapy, greater than average improvement in personality functioning can be expected.

The SF measures health-related quality of life in a total of nine different dimensions [44]. Of these, the scale “limitation in social activities because of physical or emotional problems” has the second greatest predictive power for improvement in personality functioning [44, 45]. Our results showed that patients who initially feel less restricted in social activities experience a greater improvement in personality functioning. One explanation could be that these patients experience more new relationships as a result of fewer limitations in social activities, which ‘improves’ their personality functioning [4, 46]. Together with the previous result, this finding suggests that impairments in personality functioning and limitations in social activity due to emotional and physical problems do not necessarily coincide. If patients have similar impairments in personality functioning, the patient who experiences less impairment in social activities will experience greater improvements in personality functioning.

The IIP measures interpersonal problems on a total of eight scales, which can be understood as extreme expressions of two bipolar dimensions: “Dominance” and “Affiliation”. The “too caring” scale corresponds to one pole of the “affiliation” dimension, while the “hard to be involved” and “hard to be supportive” scales correspond to the other [47, 48]. In our study, we found that patients who are too self-sacrificing or too cold towards others experience less improvement in personality functioning. Our results are therefore not entirely consistent with previous research: Ruiz et al. found that all eight scales were related to less symptomatic improvement, whereas in our study only the Affiliation dimension proved to be relevant [49]. In another study, contrary to our results, it was found that those patients who reported the most severe interpersonal problems at the start of inpatient psychotherapy experienced most symptom improvement [50]. The direction of the influence therefore remains unclear. Further, is also possible that the influence of interpersonal problems differ on symptoms and personality functioning, which could indicate that these two constructs are different. Nevertheless, the initial interpersonal problems appear to be an important predictor of improvement in personality functioning.

Previous authors looked at the influence of personality functioning on depression in the context of a diathesis-stress model [14, 51]: personality functioning is seen as a resource that protects against depressive symptoms [51]. On the other hand, an improvement in personality functioning was accompanied by more stable symptomatic improvement in follow-up measurements [14, 18-20, 52]. Our study complements these results. We found that high levels of depression and stress in the PHQ predicted greater improvement in personality functioning [53, 54]. This implies, that patients who are depressed or under greater stress also experience more improvement in their personality functioning. In consequence, these patients gain a resource that protects them from future depressive symptoms.

In contrast to Kvarstein et al., who found a negative effect of age but no effect of gender on the improvement of personality functioning, we found a greater improvement for women but no age effect [22]. Instead, we found that retired patients experience a smaller improvement in personality functioning. Thus, this is probably not an age effect, but an effect that occurs with retirement. In the study by Kvarstein et al., retirement was not included as a variable in the model [22]. Therefore, it is possible that the effect found by the colleagues is due to retirement and that there is no influence of age.

## Limitations

Our study was a retrospective observational study that used only the variables collected at the start of treatment to predict improvement in personality functioning. Further statements, such as the improvement in personality functioning depending on the length of therapy, also remain unanswerable using this approach. Many of the variables are based on self-assessment questionnaires, for some of which stronger effects were also found. This could be due to a methodological similarity in the measurement method. Furthermore, our results are limited by the fact that we had many missing values in our data set. To address this, we conducted a detailed analysis of missing values and were able to assume a ’missing at random’ process by adding some variables to our multiple imputation model. We can therefore assume that our results are unbiased, but future studies should replicate our findings [36]. Another limitation of this work is the lack of information about the therapists. Thus, we were unable to include possible relevant influencing variables, such as patient-therapist gender interactions, in our model. Whilst this is not a limitation of our study, it would be desirable to validate our model using patients outside the HIP [25, 40].

## Conclusion

We found 25 variables which can be assessed at the beginning of psychotherapy that robustly predict improvement in personality functioning during psychotherapy. These results suggest a primarily multicausal influence of other variables on improvement of personality functioning. Three noteworthy findings emerged from these results. First, patients with initially highly impaired personality functioning particularly benefit from psychotherapy. Second, limitations in social activities because of physical or emotional problems predict lower improvement in personality functioning. Third, patients who are too cold or too self-sacrificing in relationships experience less improvement in personality functioning through psychotherapy. Taken together, these findings emphasize the interpersonal and social domains as significant in the treatment of personality functioning. Clinical colleagues can collect these variables at the beginning of psychotherapy in order to develop a prognosis for treatment.

## Supporting information

Dönnhoff et al. supplementary material 1 Dönnhoff et al. supplementary material

Dönnhoff et al. supplementary material 2 Dönnhoff et al. supplementary material

Dönnhoff et al. supplementary material 3 Dönnhoff et al. supplementary material

## Data Availability

The analysis code and all results are available at doi.org/10.11588/data/50WFVL. The datasets used and analyzed during the present study cannot be shared due to restrictions by the ethical review board.

## Supplementary material

The supplementary material for this article can be found at http://doi.org/10.1192/j.eurpsy.2024.1780.
