# Supplementary material for: Predictors for improvement in personality functioning during outpatient psychotherapy: A machine learning approach within a psychodynamic psychotherapy sample
Source: Eur Psychiatry. 2024 Nov 15;67(1):e79. doi: 10.1192/j.eurpsy.2024.1780 (PMC11730054; doi:10.1192/j.eurpsy.2024.1780)
Supplement: Dönnhoff et al. supplementary material 1 — Dönnhoff et al. supplementary material [file S0924933824017802sup001.docx]

**Supplement A:**

**List of Questionnaire for the Study “Predictors for Improvement in Personality Functioning during outpatient psychotherapy: A machine learning approach within a psychodynamic psychotherapy sample”**

This is a list of all questionnaires used in our study, aside the OPD-SQ. We mention the scales and their respective scorings. We also report Mean, Standard Deviation, Minimum and Maximum of each scale as well as internal consistencies measured with Cronbach’s alpha. Patient’s answered most questionnaires directly after the clinical intake interview. Questionnaire assessing the therapeutic relationship or the therapy session were answered by both patient and therapist after the first therapy session.

*Assessment of Adverse and Protective Childhood Experiences (APC)*

The APC (Ehrenthal et al., 2020) is a self-assessment questionnaire assessing “protective childhood experiences” (PCE, M = 2.53, SD = 0.93, min = 0.25, max = 4.00, α = .95) and “adverse childhood experiences” (ACE, M = 0.80, SD = 0.61, min = 0.00, max = 3.03, α = .93). All items are answered on a 5-point Likert scale ranging from 0 = “never” to 4 = “very often”. Higher mean scores represent more protective and adverse childhood experiences, respectively.

*Beck Depression Inventory Revised (BDI-II)*

The BDI-II (Kuhner et al., 2007) is a self-assessment questionnaire assessing depressive symptoms according to ICD-10 (M = 18.85, SD = 10.44, min = 0.00, max = 56.00, α = .91). Each item measures a symptom on a four-point Likert scale, ranging from 0 = “no symptom” to 3 = “strong symptom”. The BDI-II can be used as a clinical screening instrument. The BDI-II can be used as a clinical screening instrument. A total of 18 points represents the cut-off for clinical depression. Higher sum scores represent stronger depressive symptoms.

*Depressive Experience Questionnaire (DEQ)*

The DEQ (Krieger et al., 2014) is a self-assessment questionnaire assessing two of the personality-level vulnerability factors for depression: “self-criticism” (M = 4.43, SD = 1.30, min = 1.00, max = 7.00, α = .83) and “dependency” (M = 3.97, SD = 1.45, min = 1.00, max = 7.00, α = .79). Both scales are measured using items with a 7-point Likert scale ranging from 1 = “complete rejection”, 4 = “neither” to 7 = “complete agreement”. Higher mean scores represent higher “self-criticism” and higher “dependency” respectively.

*Experience in Close Relationships – Revised (ECR-R)*

The ECR-R (Ehrenthal et al., 2009) is a self-assessment questionnaire assessing “attachment-related anxiety” (M = 3.30, SD = 1.28, min = 1.00, max = 6.44, α = .92) and “attachment-related avoidance” (M = 2.74, SD = 1.11, min = 1.00, max = 6.67, α = .91). Both scales are measured using items with a 7-point Likert scale ranging from 1 = “complete rejection”, 4 = “neither” to 7 = “complete agreement”. Higher mean scores represent higher “attachment-related anxiety” and “attachment-related avoidance”.

*Global-Assessment of Functioning Scale (GAF)*

The GAF (Heuft & Lange, 2002) is a one item rating scale (M = 24.93, SD = 8.80, min = 1.00, max = 49). The therapist is asked to assess the psychosocial and professional performance of the patient on a scale from 0 to 100. These 100 points are divided into a total of 10 areas, each of which is anchored with a description of the level of psychosocial functioning. A higher score represents overall higher psychosocial functioning. The descriptions are as follows:

100-91: Excellent performance in a wide range of activities; difficulties in life never seem to get out of control; no symptoms

90-81: No or minimal symptoms (e.g., mild anxiety before an exam), good performance in all areas, interested and involved in a wide range of activities, socially effective in behavior, generally satisfied with life, usual everyday problems or concerns (e.g., only an occasional argument with a family member)

80-71: If symptoms are present, these are temporary or expected reactions to psychosocial stress factors (e.g. difficulty concentrating after a family argument); at most slight impairment of social, occupational and academic performance (e.g. falling behind at school from time to time)

70-61: Some mild symptoms (e.g. depressed mood or mild insomnia OR some mild difficulties with social, occupational or academic performance (e.g. occasional truancy or household theft), but generally performs relatively well, has some important interpersonal relationships

60-51: Moderate symptoms (e.g. flattening of affect, rambling speech, occasional panic attacks) OR moderate difficulties with social, occupational or academic performance (e.g. few friends, conflicts with work colleagues, schoolmates or significant others)

50-41: Serious symptoms (e.g. suicidal thoughts, severe compulsive rituals, frequent shoplifting) OR impaired social, occupational and academic performance (e.g. no friends, inability to keep a job)

40-31: Some impairment in reality control or communication (e.g., speech at times illogical, incomprehensible, or irrelevant) OR severe impairment in multiple areas, e.g., work or school, family relationships, judgment, thinking, or mood (e.g., a man with depression avoids friends, neglects his family, and is unable to work; a child frequently hits younger children, is defiant at home, and fails in school

30-21: Behaviour is seriously affected by delusional phenomena or hallucinations OR serious impairment of communication and judgment (e.g., sometimes incoherent, acts grossly inadequate, strongly obsessed with suicidal thoughts OR unable to perform in almost any area (e.g., stays in bed all day, has no job, home or friends)

20-11: Danger to self and others (e.g., attempts suicide without clear intent to die, frequently violent, manic agitation) OR is occasionally unable to maintain the slightest hygiene ( e.g., smears with feces) OR grossly impaired communication (largely incoherent or mute)

10-01: Constant risk of serious injury to self or others (e.g., repeated use of force) OR persistent inability to maintain minimal personal hygiene OR serious suicide attempt with clear intent to kill.

0: Insufficient information

*Impairment Score (BSS)*

The impairment score (Schepank, 1995) is a self-assessment questionnaire assessing the severity of the impairment in the last 7 days (M = 5.19, SD = 1.80, min = 0.00, max = 12.00, α = .56) and in the last year (M = 5.85, SD = 2.03, min = 0.00, max = 12.00, α = .61). Each time frame is measured by three items assessing the bodily, mental and social impairment within a 5-point Likertscale, ranging from 0 = “no impairment” to 4 = “extreme impairment”. Higher sum scores represent higher overall impairment within the respective time frame.

*Inventory of Interpersonal Problems (IIP)*

The IIP (Barkham et al., 1996; Thomas et al., 2011) is a self-assessment questionnaire assessing 8 different types of interpersonal problems along the dimension of ‘dominance’ and ‘affiliation’: “hard to be involved” (M = 1.20, SD = 0.91, min = 0.00, max = 4.00, α = .81), “being too aggressive” (M = 0.68, SD = 0.68, min = 0.00, max = 3.75, α = .74), “being too open” (M = 1.31, SD = 0.80, min = 0.00, max = 4.00, α = .67), “being too caring” (M = 2.00, SD = 0.87, min = 0.00, max = 4.00, α = .71), “hard to be sociable” (M = 1.67, SD = 0.95, min = 0.00, max = 4.00, α = .76), “hard to be supportive” (M = 0.81, SD = 0.71, min = 0.00, max = 4.00, α = .66), “being too dependent” (M = 2.13, SD = 0.86, min = 0.00, max = 4.00, α = .71), and “hard to be assertive” (M = 2.01, SD = 0.97, min = 0.00, max = 4.00, α = .81). Each of the 8 scales is measured on a 5-point Likert scale. People are asked how much a problem applies to them. The answers range from 0 = "not at all" to 4 = "very much". Higher mean scores therefore correspond to a stronger expression in the behavioural dimensions of interpersonal problems.

*The Operationalised Psychodynamic Diagnosis Structure Questionnaire (OPD-SQ)*

The Operationalised Psychodynamic Diagnosis Structure Questionnaire was used to assess personality functioning (Ehrenthal et al., 2012). The OPD-SQ is a self-assessment questionnaire that measures the four dimensions of personality functioning (Ehrenthal et al., 2012). The four dimensions are “perception”, “regulation”, “communication”, and “attachment capacity”. All items are measured with a 5-point Likert scale ranging from 0 = “does not apply” to 4 = “does apply completely”. Higher mean scores represent greater impairments in personality functioning. Patients answered the OPD-SQ after the clinical intake interview (pre-measurement) and after the last psychotherapy session (post-measurement). Internal consistency was high: “perception/cognition” of the self (12 items, pre: M = 1.49, SD = 0.78, min = 0.00, max = 4.00, α = .88, post: M = 1.07, SD = 0.75, min = 0.00, max = 3.58, α = .91) and objects (17 items, pre: M = 1.47, SD = 0.61, min = 0.00, max = 3.65, α = .85, post: M = 1.31, SD = 0.63, min = 0.00, max = 3.47, α = .87); “regulation” of the self (13 items, pre: M = 1.53, SD = 0.66, min = 0.00, max = 3.77, α = .83, post: M = 1.20, SD = 0.67, min = 0.00, max = 3.46, α = .87) and relationships (12 items, pre: M = 1.37, SD = 0.70, min = 0.00, max = 3.70, α = .86, post: M = 1.15, SD = 0.67, min = 0.00, max = 3.55, α = .87); “communication” with the internal world (11 items, pre: M = 1.55, SD = 0.62, min = 0.00, max = 3.58, α = .75, post: M = 1.26, SD = 0.65, min = 0.00, max = 3.33, α = .84) and external world (14 items, pre: M = 1.66, SD = 0.52, min = 0.33, max = 3.13, α = .82, post: M = 1.58, SD = 0.52, min = 0.26, max = 3.47, α = .86); and “attachment capacity” to internal objects (8 items, pre: M = 1.80, SD = 0.75, min = 0.00, max = 4.00, α = .79, post: M = 1.46, SD = 0.81, min = 0.00, max = 4.00, α = .85) and external objects (8 items, pre: M = 2.28, SD = 0.77, min = 0.00, max = 4.00, α = .78, post: M = 2.05, SD = 0.79, min = 0.00, max = 3.86, α = .79).

*Outcome Questionnaire (OQ)*

The OQ (Lambert et al., 1996) is a self-assessment questionnaire designed to assess psychotherapy outcomes. It contains the scales “interpersonal relations” (M = 14.47, SD = 6.49, min = 0.00, max = 37.00, α = .77), “social role” (M = 14.10, SD = 5.19, min = 2.00, max = 29.00, α = .66) and “subjective discomfort” (M = 43.34, SD = 14.27, min = 3.00, max = 90.00, α = .90). Each item is measured on a 5-point Likert scale ranging from 0 = “never” to 4 = “almost always”. Higher sum scores thus correspond to greater stress in “interpersonal relations”, “social roles” and “subjective discomfort”.

*Patient Health Questionnaire (PHQ)*

The PHQ (Gräfe et al., 2004; Löwe et al., 2002) is a self-assessment screening instrument for mental health disorders. In this study, the scales “anxiety” (M = 8.38, SD = 4.41, min = 0.00, max = 21.00, α = .83), “depression” (M = 10.68, SD = 5.58, min = 0.00, max = 27.00, α = .85), “somatic symptoms” (M = 10.78, SD = ,5.61 min = 0.00, max = 30.00, α = .78), and “stress” (M = 7.29, SD = 3.82, min = 0.00, max = 18.00, α = .65) were used. The scales “anxiety” and “depression” are measured on a 4-point Likert scale ranging from 0 = “none” to 3 = “nearly every day”. Thus, higher sum scores represent more depressive and anxious symptoms. The scales “somatic symptoms” and “stress” are measured on a 3-point Likert scale ranging from 0 = “no impairment” to 2 = “strong impairment”. Therefore, higher sum scores represent stronger impairment by “stress” and “somatic symptoms” respectively.

*Session Evaluation questionnaire (SEQ)*

The SEQ (Stiles, 1980; Stiles et al., 1994) is a self-assessment questionnaire proposed as a measure of session impact with both a version for patients (SEP) and therapists (SET). It assesses two scales: “depth” (patient: M = 4.91, SD = 0.88, min = 2.00, max = 7.00, α = .66, therapist: M = 4.51, SD = 0.77, min = 1.60, max = 6.80, α = .72) and “smoothness” (patient: M = 4.75, SD = 1.03, min = 2.20, max = 7.00, α = .74, therapist: M = 4.19, SD = 1.03, min = 1.20, max = 7.00, α = .87). Each item of both scales is measured on a 7-point Likert scale. Hereby, “1” corresponds to the most negative and 7 to the most positive answer for the corresponding item. Therefore, higher mean scores represent more “depth” and “smoothness” within the first psychotherapy session.

*Short-Form-36 Health Survey (SF)*

The SF (Bullinger, 2000; Bullinger & Kirchberger, 1998; Bullinger et al., 1995) is a generic instrument for the self-assessment of health-related quality of life. Its nine scales consist of “general health perception” (M = 42.04, SD = 12.35, min = 0.00, max = 80.00, α = .78), “limitations in usual role activities because of emotional problems” (M = 37.62, SD = 39.22, min = 0.00, max = 100.00, α = .76), “limitations in physical activities because of health problems” (M = 85.72, SD = 18.45, min = 10.00, max = 100.00, α = .91), “limitations in usual role activities because of physical health problems” (M = 60.62, SD = 39.21, min = 0.00, max = 100.00, α = .83), “bodily pain” (M = 61.94, SD = 30.10, min = 0.00, max = 100.00, α = .85), “general mental health” (M = 46.56, SD = 17.11, min = 0.00, max = 96.00, α = .81), “limitations in social activities because of physical or emotional problems” (M = 55.81, SD = 26.32, min = 0.00, max = 100.00, α = .71), “change in health” (only one item, M = 39.60, SD = 27.57, min = 0.00, max = 100.00), and “vitality” (M = 34.92, SD = 18.94, min = 0.00, max = 90.00, α = .86). Each item of the SF-36 has its own response format. The answer that expresses the greatest health has a value of 100, while the answer that expresses the greatest impairment or illness has a value of 0. In contrast to almost all other questionnaires and the naming of some scales, higher scores on a scale therefore correspond to greater health and fewer impairments for the patient.

*Symptom-Checklist-K11 (SCL-K11)*

The SCL-K11 (Müller et al., 2009) is a self-assessment questionnaire assessing general psychopathology (M = 1.23, SD = 0.74, min = 0.00, max = 3.80, α = .87). The 11 items are answered on a 5-point Likert scale ranging from 0 = “not at all” to 4 = “very strongly”. Thus, higher mean scores represent higher symptom severity.

*Working Alliance Inventory (WAI)*

The WAI (Busseri & Tyler, 2003; Horvath & Greenberg, 1989; Munder et al., 2010; Wilmers et al., 2008) is a self-assessment questionnaire assessing the therapeutic alliance. Both patient (WAP) and therapist (WAT) are asked about agreement on the “tasks” of therapy (patient: M = 3.36, SD = 0,80, min = 1.00, max = 5.00, α = .80, therapist: M = 3.03, SD = 0.65, min = 1.00, max = 4.80, α = .83), agreement on the “goals” of therapy (patient: M = 3.77, SD = 0.86, min = 1.00, max = 5.00, α = .83, therapist: M = 3.23, SD = 0.71, min = 1.00, max = 5.00, α = .86), and development of an affective “bond” (patient: M = 4.07, SD = 0.72, min = 1.25, max = 5.00, α = .75, therapist: M = 3.83, SD = 0.58, min = 2.00, max = 5.00, α = .87). All items of all scales are measured on a 5-point Likert scale, ranging from 1 = "Rarely" to 5 = "Always". This applies to both the patient and therapist versions of the questionnaire. Higher mean values therefore correspond to a better therapeutic relationship.

**Sources**

Barkham, M., Hardy, G. E., & Startup, M. (1996). The IIP‐32: A short version of the Inventory of Interpersonal Problems. *British Journal of Clinical Psychology*, *35*(1), 21-35.

Bullinger, M. (2000). Erfassung der gesundheitsbezogenen Lebensqualität mit dem SF-36-Health Survey. *Bundesgesundheitsblatt-Gesundheitsforschung-Gesundheitsschutz*, *43*(3), 190-197.

Bullinger, M., & Kirchberger, I. (1998). SF-36-Fragebogen zum Gesundheitszustand (PSYNDEX Tests Review). *MOS Short-Form-36 Health Survey (SF-36*.

Bullinger, M., Kirchberger, I., & Ware, J. (1995). Der deutsche SF-36 Health Survey Übersetzung und psychometrische Testung eines krankheitsübergreifenden Instruments zur Erfassung der gesundheitsbezogenen Lebensqualität. *Zeitschrift für Gesundheitswissenschaften= Journal of public health*, *3*(1), 21-36.

Busseri, M. A., & Tyler, J. D. (2003). Interchangeability of the Working Alliance Inventory and Working Alliance Inventory, Short Form. *Psychol Assess*, *15*(2), 193-197. <https://doi.org/10.1037/1040-3590.15.2.193>

Ehrenthal, Dinger, U., Horsch, L., Komo-Lang, M., Klinkerfuss, M., Grande, T., & Schauenburg, H. (2012). The OPD Structure Questionnaire (OPD-SQ): first results on reliability and validity. *Psychotherapie, Psychosomatik, Medizinische Psychologie*, *62*(1), 25-32.

Ehrenthal, Schauenburg, H., Wagner, F. E., Dinger, U., & Volz, M. (2020). [Development and Evaluation of the Questionnaire for the Assessment of Adverse and Protective Childhood Experiences (APC)]. *Psychiatr Prax*, *47*(4), 207-213. <https://doi.org/10.1055/a-1123-1615>

Ehrenthal, J. C., Dinger, U., Lamla, A., Funken, B., & Schauenburg, H. (2009). [Evaluation of the German version of the attachment questionnaire "Experiences in Close Relationships--Revised" (ECR-RD)]. *Psychother Psychosom Med Psychol*, *59*(6), 215-223. <https://doi.org/10.1055/s-2008-1067425> (Evaluation der deutschsprachigen Version des Bindungsfragebogens "Experiences in Close Relationships--Revised" (ECR-RD).)

Gräfe, K., Zipfel, S., Herzog, W., & Löwe, B. (2004). Screening psychischer Störungen mit dem “Gesundheitsfragebogen für Patienten (PHQ-D)“. *Diagnostica*, *50*(4), 171-181. <https://doi.org/10.1026/0012-1924.50.4.171>

Heuft, G., & Lange, C. (2002). Die Beeinträchtigungsschwere in der psychosomatischen und psychiatrischen Qualitätssicherung: Global Assessment of Functioning Scale (GAF) vs. Beeinträchtigungs-Schwere-Score (BSS). *Zeitschrift für Psychosomatische Medizin und Psychotherapie*, *48*(3), 256-269.

Horvath, A. O., & Greenberg, L. S. (1989). Development and validation of the Working Alliance Inventory. *Journal of Counseling Psychology*, *36*(2), 223.

Krieger, T., Zimmermann, J., Beutel, M. E., Wiltink, J., Schauenburg, H., & Holtforth, M. G. (2014). Ein Vergleich verschiedener Kurzversionen des Depressive Experiences Questionnaire (DEQ) zur Erhebung von Selbstkritik und Abhängigkeit. *Diagnostica*, *60*(3), 126-139. <https://doi.org/10.1026/0012-1924/a000105>

Kuhner, C., Burger, C., Keller, F., & Hautzinger, M. (2007). [Reliability and validity of the Revised Beck Depression Inventory (BDI-II). Results from German samples]. *Nervenarzt*, *78*(6), 651-656. <https://doi.org/10.1007/s00115-006-2098-7> (Reliabilitat und Validitat des revidierten Beck-Depressionsinventars (BDI-II). Befunde aus deutschsprachigen Stichproben.)

Lambert, M. J., Burlingame, G. M., Umphress, V., Hansen, N. B., Vermeersch, D. A., Clouse, G. C., & Yanchar, S. C. (1996). The reliability and validity of the outcome questionnaire. *Clinical Psychology & Psychotherapy*, *3*(4), 249-258. <https://doi.org/10.1002/(SICI)1099-0879(199612)3:4><249::AID-CPP106>3.3.CO;2-J

Löwe, B., Spitzer, R., Zipfel, S., & Herzog, W. (2002). Autorisierte deutsche Version des “Prime MD Patient Health Questionnaire (PHQ)”. *Auflage. New York: Pfizer*.

Müller, J. M., Postert, C., Beyer, T., Furniss, T., & Achtergarde, S. (2009). Comparison of Eleven Short Versions of the Symptom Checklist 90-Revised (SCL-90-R) for Use in the Assessment of General Psychopathology. *Journal of Psychopathology and Behavioral Assessment*, *32*(2), 246-254. <https://doi.org/10.1007/s10862-009-9141-5>

Munder, T., Wilmers, F., Leonhart, R., Linster, H. W., & Barth, J. (2010). Working Alliance Inventory-Short Revised (WAI-SR): psychometric properties in outpatients and inpatients. *Clin Psychol Psychother*, *17*(3), 231-239. <https://doi.org/10.1002/cpp.658>

Schepank, H. (1995). BSS–Der Beeinträchtigungs-Schwere-Score. *Göttingen: Beltz-Test*.

Stiles, W. B. (1980). Measurement of the impact of psychotherapy sessions. *Journal of Consulting and Clinical Psychology*, *48*(2), 176.

Stiles, W. B., Reynolds, S., Hardy, G. E., Rees, A., Barkham, M., & Shapiro, D. A. (1994). Evaluation and description of psychotherapy sessions by clients using the Session Evaluation Questionnaire and the Session Impacts Scale. *Journal of Counseling Psychology*, *41*(2), 175.

Thomas, A., Brähler, E., & Strauß, B. (2011). IIP-32: Entwicklung, Validierung und Normierung einer Kurzform des Inventars zur Erfassung interpersonaler Probleme. *Diagnostica*, *57*(2), 68-83. <https://doi.org/10.1026/0012-1924/a000034>

Wilmers, F., Munder, T., Leonhart, R., Herzog, T., Plassmann, R., Barth, J., & Linster, H. W. (2008). Die deutschsprachige Version des Working Alliance Inventory-short revised (WAI-SR)-Ein schulenübergreifendes, ökonomisches und empirisch validiertes Instrument zur Erfassung der therapeutischen Allianz. *Klinische Diagnostik und Evaluation*, *1*(3), 343-358.
