# Supplementary material for: Predictors for improvement in personality functioning during outpatient psychotherapy: A machine learning approach within a psychodynamic psychotherapy sample
Source: Eur Psychiatry. 2024 Nov 15;67(1):e79. doi: 10.1192/j.eurpsy.2024.1780 (PMC11730054; doi:10.1192/j.eurpsy.2024.1780)
Supplement: Dönnhoff et al. supplementary material 2 — Dönnhoff et al. supplementary material [file S0924933824017802sup002.docx]

**Tabular comparison of the dimensions of Personality Functioning from OPD-2, AMPD of the DSM-5, ICD-11**

A comparison of the manuals shows that the AMPD of the DSM-5 and the ICD-11 place a greater emphasis on the pursuit of goals and decision-making processes [1-4]. These elements are not explicitly included in the OPD-2 [3, 5]. OPD-2, on the other hand, focuses more on internal psychological processes such as fantasy and pleasure, but also on previous experiences with other people, which are defined as introjects or representations. OPD-2 also looks at people's ability to triangulate. In other words, the ability to take on new experiences and relationships with existing ones and also to let go of existing relationships and introjects. All these constructs play an important role in psychoanalytic theory and treatment and are therefore dealt with here. The DSM-5 and ICD-11 lack a counterpart here, as they focus on explicitly observable behaviour [1, 3, 4, 6].

The following table provides a tabular overview of the elements of personality functioning comparing the approaches of OPD-2, DSM-5 and ICD-11. We have decided not to use the order of the dimensions in the respective manual. Instead, we have first listed the elements that belong to the "Self" dimension according to ICD-11 and then the elements that belong to the "Interpersonal" dimension. Furthermore, the elements are arranged in such a way that elements that are close to each other in terms of content are also arranged at the same height. For the OPD-2 elements we included both the name of the dimension each element is attributed to as well as the psychoanalytic terminology (if one exists) of the element. For the DSM-5 we also included the name of the dimension an element is attributed to.

We would like to add, that some element of one might correspond to several other elements of other manuals. In some cases we deemed it appropriate to correspond one element to multiple others. Other categorisations are therefore also conceivable.

| ***Self*** | | |
| --- | --- | --- |
| **OPD-2** | **DSM-5** | **ICD-11** |
| Perception: Reflect and differentiate self image | Identity: accuracy of one’s self-assessment | Accuracy of one’s view of one’s characteristics, strengths, limitations |
|  |  | Stability and coherence of one’s sense of identity (e.g. extent to which identity or sense of self is variable and inconsistent or overly rigid and fixed) |
|  | Self-direction: Ability of productive self-reflection |  |
| Perception: Differentiate one’s own affects |  | Ability to recognize and acknowledge emotions that are difficult or unwanted by the individual (e.g. anger, sadness) |
| Perception: Design and further develop one’s own identity | Identity: Perceive oneself as unique, with clear boundaries between oneself and others |  |
| Regulation: Distance oneself from impulses, controlling and integrating impulses |  | Flexibility in controlling impulses and modulating behaviour based on the situation and consideration of the consequences |
|  |  | Tendency to be emotionally over- or underreactive |
|  | Self-direction: Pursue coherent and meaningful short-term or long-term goals. | Capacity for self-direction (ability to plan, choose, and implement appropriate goals) |
| Regulation: Distance oneself from affects, regulate affects | Identity: Ability to experience and regulate a wealth of emotions | Flexibility in controlling impulses and modulating behaviour based on the situation and consideration of the consequences |
|  |  | Tendency to be emotionally over- or underreactive |
| Regulation: Distance oneself from emotional hurts, regulate self-worth | Identity: Stability of one’s self-worth | Ability to maintain an overall positive and stable sense of self-worth |
| Communication: Generate and experience one’s own affects | Identity: Ability to experience and regulate a wealth of emotions | Range and appropriateness of emotional experience and expression |
| Communication: Create and use one’s own fantasies |  |  |
| Communication: Emotionally animate the perception of one’s own body or bodily self |  | Appropriateness of behavioural responses to intense emotions and stressful circumstances (e.g. propensity to self-harm or violence) |
| Attachment capacity: Internalization. Positive self-representations, positive object-representations, ability to build and maintain positive object-related affects |  |  |
| Attachment capacity: Positive introjects. Ability to care for oneself, to calm, console, help, protect oneself, to stand in for oneself |  | Ability to manage conflict in relationships |
| Attachment capacity: Variable and triangular attachments. Different internal object qualities; attachment to one does not mean turning away from another |  |  |
|  | Self-direction: Orientation towards constructive and prosocial standards of behavior |  |
|  |  | Ability to make appropriate decisions in situations of uncertainty |
|  |  | Appropriate stability and flexibility of belief systems |

| ***Interpersonal and Relationships*** | | |
| --- | --- | --- |
| **OPD-2** | **DSM-5** | **ICD-11** |
| Perception: Self/object differentiation. Distinguish one’s own thoughts, needs, impulses from those of others |  |  |
| Perception: Perceive others in their various aspects, that is as whole persons | Empathy: Understanding and recognizing the experiences and motives of others | Ability to understand and appreciate others’ perspectives |
| Perception: Ability to design a realistic picture of others | Empathy: Understanding and recognizing the experiences and motives of others | Ability to understand and appreciate others’ perspectives |
| Regulation: Protect the relationship from one’s own disturbing elements; intrpsychic instead of interpersonal defense | Empathy: Understanding the effect of your own behavior | Ability to develop and maintain close and mutually satisfying relationships |
| Regulation: In relationships, maintain one’s own interests and take due account of the interests of others | Empathy: Tolerance towards different points of view |  |
| Regulation: Ability to develop a realistic picture of others | Empathy: Understanding and recognizing the experiences and motives of others | Accuracy of situational and interpersonal appraisals, especially under stress |
| Communication: Make emotional contact. Allow feelings towards others, dare to make emotional investments, achieve “we” feeling (reciprocity) | Intimicy: Depth and duration of (positive) relationship with others | Ability to develop and maintain close and mutually satisfying relationships |
| Communication: Express one’s own affects, let oneself be reached by the affects of others |  |  |
| Communication: Experience empathy | Empathy |  |
| Attachment capacity: Ability to make attachments. Attach others emotionally (gratitude, loving care, guilt, sadness) | Intimicy: Desire and ability to be close to others | Interest in engaging in relationships with others |
| Attachment capacity: Accepting help. Ability to accept support, care, concern, guidance, apologies from others |  |  |
|  | Intimicy: Mutual respect, which is reflected in interpersonal behavior |  |
| Attachment capacity: Ability to sever attachments and tolerate farewells |  | Ability to manage conflict in relationships |

Sources

1. APA. Diagnostic and statistical manual of mental disorders2022.

2. WHO. International Classification of Diseases 2021 [26.04.2024]. Available from: <https://icd.who.int/en>.

3. Blüml V, Doering S. ICD-11 Personality Disorders: A Psychodynamic Perspective on Personality Functioning. Frontiers in Psychiatry. 2021;12.

4. Organization WH. Clinical descriptions and diagnostic requirements for ICD-11 mental, behavioural and neurodevelopmental disorders (CDDR): World Health Organization; 2024 [updated 08.03.2024.

5. Cierpka M, editor. Operationalisierte Psychodynamische Diagnostik OPD-2 : das Manual für Diagnostik und Therapieplanung. 1. Aufl. ed. Bern: Huber; 2006.

6. Bender DS, Morey LC, Skodol AE. Toward a Model for Assessing Level of Personality Functioning in DSM–5, Part I: A Review of Theory and Methods. Journal of Personality Assessment. 2011;93(4):332-46.
